# Plant Tissues as 3D Natural Scaffolds for Adipose, Bone and Tendon Tissue Regeneration

**DOI:** 10.3389/fbioe.2020.00723

**Published:** 2020-06-30

**Authors:** Nicola Contessi Negrini, Nadia Toffoletto, Silvia Farè, Lina Altomare

**Affiliations:** ^1^Department of Chemistry, Materials and Chemical Engineering “G. Natta”, Politecnico di Milano, Milan, Italy; ^2^National Interuniversity Consortium of Materials Science and Technology, Local Unit Politecnico di Milano, Milan, Italy

**Keywords:** plant tissues, decellularization, adipose tissue engineering, bone tissue engineering, tendon tissue engineering

## Abstract

Decellularized tissues are a valid alternative as tissue engineering scaffolds, thanks to the three-dimensional structure that mimics native tissues to be regenerated and the biomimetic microenvironment for cells and tissues growth. Despite decellularized animal tissues have long been used, plant tissue decellularized scaffolds might overcome availability issues, high costs and ethical concerns related to the use of animal sources. The wide range of features covered by different plants offers a unique opportunity for the development of tissue-specific scaffolds, depending on the morphological, physical and mechanical peculiarities of each plant. Herein, three different plant tissues (i.e., apple, carrot, and celery) were decellularized and, according to their peculiar properties (i.e., porosity, mechanical properties), addressed to regeneration of adipose tissue, bone tissue and tendons, respectively. Decellularized apple, carrot and celery maintained their porous structure, with pores ranging from 70 to 420 μm, depending on the plant source, and were stable in PBS at 37°C up to 7 weeks. Different mechanical properties (i.e., E_apple_ = 4 kPa, E_carrot_ = 43 kPa, E_celery_ = 590 kPa) were measured and no indirect cytotoxic effects were demonstrated *in vitro* after plants decellularization. After coating with poly-L-lysine, apples supported 3T3-L1 preadipocytes adhesion, proliferation and adipogenic differentiation; carrots supported MC3T3-E1 pre-osteoblasts adhesion, proliferation and osteogenic differentiation; celery supported L929 cells adhesion, proliferation and guided anisotropic cells orientation. The versatile features of decellularized plant tissues and their potential for the regeneration of different tissues are proved in this work.

## Introduction

Tissue engineering requires an accurate design of engineered biomaterials able to sustain the regeneration of pathological/missing tissues. Both synthetic and natural-derived biomaterials have been proposed and are still under investigation to achieve the appropriate morphological, physical, mechanical and biological properties to suit the specific requirements for the regeneration of different target human tissues. Synthetic polymer-based scaffolds are reproducible, possess a defined chemical composition and tuneable properties according to the application requirements. Natural-derived polymer-scaffolds, despite the limited reproducibility, are characterized by a superior biological response compared to synthetic scaffolds, a good biocompatibility and ecological safety (Alaribe et al., 2016). However, in both cases, the polymeric materials must be processed to fabricate scaffolds with the appropriate properties and, despite advanced fabrication technologies have been developed to process polymers, reproducing the correct microarchitecture of the tissues to be regenerated is still challenging (Jammalamadaka and Tappa, 2018).

Decellularization of tissues offers a valid alternative to avoid materials’ processing since the biomimetic native structure can be preserved while removing the cellular components to obtain three-dimensional acellular scaffolds for tissue engineering applications (Urciuolo et al., 2018). Decellularized matrices are a suitable morphological and biochemical microenvironment for cell adhesion, proliferation and differentiation (Wu et al., 2015). Moreover, compared to allogeneic and xenogeneic transplant, decellularized structures have lower immunogenic response in the host after implantation, as immunogenicity is associated to intracellular components that are removed by the decellularization process.

Different animal-derived tissues were decellularized to obtain extracellular matrix (ECM) scaffolds with a preserved vascular structure for the transfer of oxygen and nutrients, including skin, (Farrokhi et al., 2018) bone (Smith et al., 2017) and vascular grafts (Lin et al., 2018). Some of these acellular patches are now commercially available, such as Alloderm,^®^ ReadiGRAFT,^®^ OrACELL,^®^ and ArthroFLEX^®^. In the last decade, decellularization of whole organs also gained an increasing interest [e.g., acellular heart, (Kitahara et al., 2016) lungs, (Crabbé et al., 2015) kidney, (Xue et al., 2018) liver, (Bühler et al., 2015), and pancreas (Guruswamy Damodaran and Vermette, 2018) from animal or human donors] and a partial organ functionality was successfully restored after cell recolonization (Petersen et al., 2010). Despite these promising results, animal and human sources are controversial and affected by limited availability, high production costs and ethical concerns (Porzionato et al., 2018). Moreover, the variability among different donors (e.g., age, pathology, explant site) (Song and Ott, 2011) is a critical aspect in clinical applications and the use of animal sources may lead to a host reaction to xenoantigens (Scarrit, 2015) or the transmission of infectious agents (Gilbert et al., 2006).

Very recently, plant-derived tissues gained tremendous interest (Gershlak et al., 2017; Phan et al., 2020) as an alternative to animal sources to obtain decellularized scaffolds for tissue engineering applications. Since a wide variety of plant architectures exists in the plant kingdom, decellularized plant-derived scaffolds could be selected depending on their native structure and properties to mimic a multiplicity of mammalian tissues (Gershlak et al., 2017). In fact, in addition to their readily availability, low cost, ease of use, and absence of ethical issues, plant tissues exhibit good cytocompatibility (Modulevsky et al., 2014) and biocompatibility (Modulevsky et al., 2016). For instance, 2D scaffolds were obtained after decellularization of leaves [e.g., Artemisia annua, (Gershlak et al., 2017) Anthurium, (Fontana et al., 2017) Ficus hispida, (Adamski et al., 2018) spinach leaves] (Gershlak et al., 2017; Dikici et al., 2019) by preserving their branched vessels, resembling mammalian vasculature. On decellularized spinach leaves, (Gershlak et al., 2017) a contractile function and calcium handling capabilities were observed after 21 days of cardiomyocytes culture, confirming the suitability of the scaffold for *in vitro* cardiac tissue regeneration. Tubular scaffolds resulting from parsley, bamboo and vanilla stems (Fontana et al., 2017) were successfully decellularized and an efficient cell proliferation was observed after recellularization. Three-dimensional plant structures were also obtained by decellularization procedures from green onion bulb (Cheng et al., 2020) and apple hypanthium (Modulevsky et al., 2014, 2016; Hickey et al., 2018; Lee et al., 2019). A similar strategy was adopted for fungi tissues to obtain decellularized mushroom caps (Balasundari et al., 2012). Decellularized apple scaffolds, characterized by an open porosity, provided media transfer and supported mammalian cell growth up to 12 weeks of *in vitro* culture (Modulevsky et al., 2014). The *in vivo* implantation of apple-derived scaffolds resulted in the growth of functional blood vessels in the material after 8 weeks (Modulevsky et al., 2016; Lee et al., 2019). Thus, the pro-angiogenic features of scaffolds obtained from vegetables and their interconnected porous structures make them potential candidates for the proliferation and survival of cells and tissues growth.

Despite the advantages offered by decellularized plant-derived scaffolds and the promising results obtained so far, the versatility of these structures in terms of tissues to be potentially regenerated has not been fully investigated yet. The wide range of features covered by different plants (i.e., morphology, structure, cell-instructive properties) constitutes a unique alternative for the development of tissue-engineered scaffolds, and the peculiarities of selected plants potentially matching the requirements for the regeneration of specific human body tissues has still to be fully explored. In this context, we identified three different plant tissues (i.e., apple, carrot, and celery) that are characterized by diverse internal architectures. Then, according to the obtained morphological, physical and mechanical characterization, we selected specific applications for each plant and investigated the *in vitro* potential as scaffolds for the regeneration of different tissues (i.e., adipose tissue, bone tissue and tendons, respectively).

## Materials and Methods

All reagents were purchased from Sigma Aldrich, unless differently specified. Fresh plants were acquired from the same chain store and stored at 4°C, for a maximum of 2 days, prior to use.

### Plant Tissues Preparation and Decellularization

**FIGURE 1:**
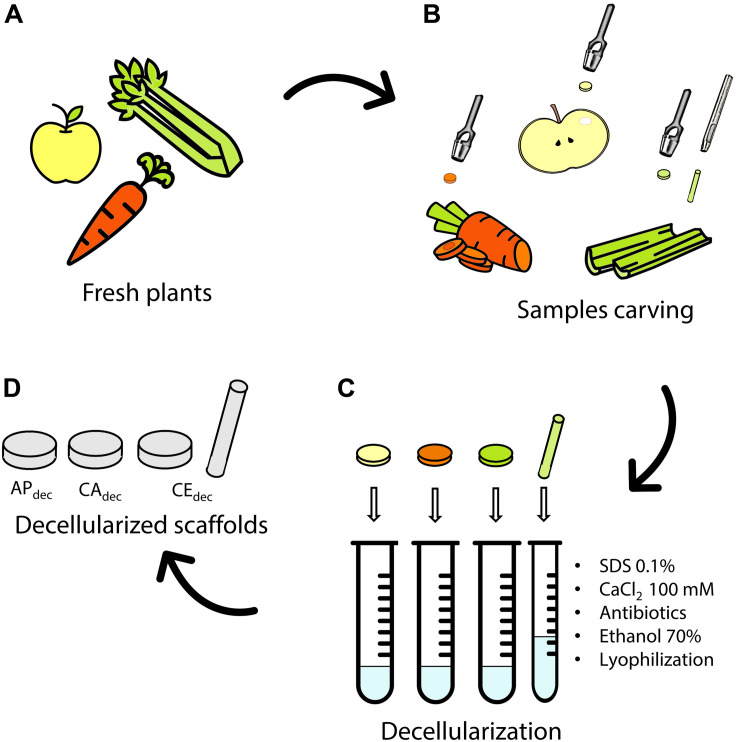
Decellularization of plant tissues. **(A)** Selected fresh plants are **(B)** carved by a biopsy punch. **(C)** Samples are decellularized by subsequent immersion in decellularization/washing solution to obtain **(D)** apple (AP_dec_), carrot (CA_dec_) and celery (CE_dec_) scaffolds. Icons The Noun Project.

Three different plant tissues (Figure 1A) were selected and tested as potential scaffolds for human tissues regeneration: Golden Delicious apple (*Malus domestica)*, carrot (*Daucus carota*), and celery (*Apium graveolens*). Slices (thickness *t* = 2 mm) were cut with a mandolin slicer. Cylindrical samples (diameter Ø = 10 mm) were then carved by biopsy punch (Figure 1B). Apple specimens (AP_dec_) were carved from the outer hypanthium tissue. Carrot specimens (CA_dec_) were obtained by punching the xylem from the transversal section. Celery samples (CE_dec_) were sliced in longitudinal direction and punched selecting the stem pith. CE samples (Ø = 4 mm, length *L* = 25 mm) were also carved in longitudinal direction to be used for mechanical testing.

Decellularization (Figure 1C) was performed according to a previously established protocol for plant tissues (Hickey et al., 2018). Briefly, each sample was immersed 5 ml of 0.1% w/v sodium dodecyl sulfate (SDS) solution for 48 h at room temperature under continuous shaking at 180 rpm. After 24 h, samples were sonicated for 5 min at 40°C and the SDS solution was renewed. Samples were then washed three times in distilled water and washed in 100 mM CaCl_2_ for 24 h. Control samples (AP_ctr_, CA_ctr_, and CE_ctr_) were freshly cut from the plants following the previously described procedure and were not treated by the described decellularization process. Both decellularized and control samples were washed three times in distilled water and subsequently incubated with 1% w/v penicillin/streptomycin and 1% w/v amphotericin B for 3 h under shaking at 140 rpm. Finally, samples were disinfected in 70% v/v ethanol solution for 1 h, washed three times in sterile distilled water and frozen at −20°C overnight. The obtained structures were then lyophilized (Freeze drier Lio 5Pascal; −40°C, 24 h, *P* < 0.5 mbar) and sterilized by UV irradiation for 15 min on each side.

### Characterization of Decellularized Plant Tissues

#### Morphological Characterization

The morphology of decellularized plant tissues was investigated by stereomicroscopy (Wild M8 stereomicroscope and Leica LAS core software, 6× magnification) and compared with fresh-cut samples. Scanning Electron Microscopy (SEM, Cambridge StereoScan 360) analysis was conducted on gold-sputter coated samples to observe pore morphology and size in both decellularized and control samples. The analysis was performed in secondary electron mode (10 kV), at 15 and 100× magnification. Celery samples were observed in both longitudinal and transversal direction to investigate the anisotropic structure. From the obtained images, the average pore size was calculated according to ASTM D3576 – 15 standard; results are expressed as mean ± inter-image variability (*n* = 3).

#### Physical Characterization

The dimension of the scaffolds (thickness and diameter) was measured by a caliper on fresh-cut samples (*n* = 4). Then, samples were measured, after decellularization, in the hydrated state and results were compared to the dimension of fresh-cut samples to investigate possible volumetric changes (i.e., percentage shrinkage) due to the decellularization treatment.

Water absorption and retention over time were evaluated by soaking decellularized and control dehydrated samples (*n* = 4) in phosphate buffered saline (PBS) with 0.02% sodium azide (used as bacteriostatic) at 37°C, up to 7 weeks. The mass of anhydrous samples was measured (w_0_) and samples were weighted (w_t_) at established time points (*t* = 1, 2, 3, 4, 5, 6, 24, 48, 72 h, and weekly for 7 weeks), after gentle swabbing to remove excess water from the surface. The water uptake Δw[%] at the different time points was then calculated using Eq. 1:

(1) $$\Delta w\left[\%\right]=\frac{w_{t}-w_{0}}{w_{t}}\times 100$$

#### *In vitro* Indirect Cytotoxicity Test

Indirect cytotoxicity test was performed *in vitro* according to the standard practice EN ISO 10993-5, to evaluate the effect of possible release of cytotoxic products resulting from the decellularization procedure or from the scaffolds’ composition. L929 murine fibroblast cells (ECACC No 85011425) were seeded in 96-well tissue culture polystyrene (TCPS) plates (1 × 10^4^ cells per well) and cultured (i.e., culture medium M_fibr_: Dulbecco’s Modified Eagle’s Medium, DMEM, added with 10% fetal bovine serum, 1 mM sodium pyruvate, 1% penicillin-streptomycin, 10 mM 4(2-hydroxyethyl)-1-piperazine ethanesulfonic acid and 4 mM L-glutamine) at 37°C in 5% CO_2_ humidified atmosphere, until 70% confluence was reached. Eluates were obtained by incubating decellularized samples (*n* = 3 per type, per time point) in the culture medium for 1, 4, and 7 days. The volume of incubation medium was adapted from EN ISO 10993-12. At each time point, culture medium was also incubated without samples, as control. Cells were then cultured for 24 h with culture medium eluates or culture medium controls. Cell viability was assessed by alamarBlue assay; culture medium was replaced by 10% v/v alamarBlue solution in fresh culture medium and cells were incubated for 3 h. Then, 100 μl of surnatants were transferred from each well to a new 96-well TCPS, in triplicate, and fluorescence was read by a spectrophotometer (GENios Plus Reader, TECAN; λ_exc_ = 540 nm, λ_em_ = 595 nm). The percentage cell viability was calculated as ratio between the fluorescence values measured for cells cultured with eluates (*f*_*eluates*_) to the fluorescence values obtained from cells cultured in culture medium controls (*f*_*control*_), after subtracting the background of the alamarBlue solution (*f*_*AlamarBlue*_), as for Eq. 2:

(2) $$Cellviability\left[\%\right]=\frac{f_{e\,l\,u\,a\,t\,e\,s}-f_{A\,l\,a\,m\,a\,r\,B\,l\,u\,e}}{f_{c\,o\,n\,t\,r\,o\,l}-f_{A\,l\,a\,m\,a\,r\,B\,l\,u\,e}}\times 100$$

### Apple-Derived Scaffolds for Adipose Tissue Engineering

#### Mechanical Properties

The compression properties of apple-derived scaffolds were tested by Dynamic Mechanical Analyzer (DMA Q800, TA instruments). Control and decellularized apple samples (*n* = 4, Ø = 10 mm, *t* = 2 mm) were soaked in PBS at 37°C for 1 week (i.e., Δw plateau) and then tested in the swollen state by applying one hysteresis compression cycle. Each test consisted in a load phase at a rate of 2.5% min^–1^ down to −30% strain, as previously reported for adipose tissue engineering, (Chang et al., 2013; Contessi Negrini et al., 2020) and subsequent unload phase at a rate of 5% min^–1^. From the stress-strain curves, the elastic modulus E (calculated as the slope in the 0–5% strain range), stiffness k (slope in the 15–20% strain range), maximum stress σ_max_, residual strain ε_res_ and hysteresis area H (calculated as the area between the load and unload curves) were calculated.

#### *In vitro* Direct Cytocompatibility Test

Decellularized apple scaffolds (*n* = 4) were lodged into 24-well TCPS plates and coated to promote cell adhesion by incubation in 0.01% w/v poly-L-lysine (PLL) overnight, with gentle agitation (50 rpm) at 37°C, then washed 3 times with PBS to remove the excess of PLL and transferred to new plates. 3T3-L1 murine cell line (ECACC No 86052701) was selected as *in vitro* model for adipose tissue engineering. Cells were seeded on decellularized apple samples (2 × 10^5^ cells per sample) and on 24-well TCPS plates, as control, and incubated (37°C, 5% CO_2_) for 20 min to promote cell adhesion. Then, 1.2 ml of preadipocyte growth medium (M_preadipo_: DMEM with 10% v/v fetal calf serum, 10 mM HEPES, 4 mM L-glutamine, 1 mM sodium pyruvate and 1% penicillin – streptomycin) was added to culture cells by renewing the medium every 2 days. After 7 days of culture, adipogenic differentiation was induced by culture in differentiation culture medium for 48 h (M_diff_: DMEM with 10% v/v fetal bovine serum, 10 mM HEPES, 4 mM L-glutamine, 1 mM sodium pyruvate, 1% penicillin-streptomycin, 1 μg ml^–1^ insulin, 0.5 mM 3-isobutyl-1-methylxanthine (IBMX), 1 μM dexamethasone (DEX) and 1 μM rosiglitazone) (Zebisch et al., 2012). Then, differentiation-induced samples (AP_adipo_) and wells (TCPS_adipo_) were kept in maintenance medium up to 14 days of culture, renewed each 2 days (M_maint_: composed by M_diff_ without IBMX, DEX and rosiglitazone). As control (i.e., no adipogenesis induced), seeded samples (AP_preadipo_) and wells (TCPS_preadipo_) were kept in preadipocyte growth medium for the whole duration of the test, by renewing culture medium every 2 days.

Metabolic activity of cells cultured on apple-derived scaffolds and TCPS wells (*n* = 4) was investigated by alamarBlue assay after 1, 3, 7, 10, and 14 days of culture. At each time point, scaffolds were transferred to new plates to discharge cells on the well bottom. Samples were incubated in 1.2 ml of 10% v/v alamarBlue solution in culture medium for 4 h; then 100 μl of eluates were transferred in triplicate in a 96-well plate and fluorescence was read as previously described. Samples were rinsed twice with PBS and incubated with culture medium (M_preadipo_, M_diff_ or M_maint_) until the subsequent time point. After 7 days of culture, a LIVE/DEAD staining was performed on scaffolds (*n* = 3) to qualitatively investigate the distribution of viable and dead cell. Samples were incubated in the staining solution (10 μM propidium iodide and 2 μM calcein-AM in PBS) for 40 min, washed three times with PBS and immersed in culture medium without FBS. Images (*n* = 7) were acquired by fluorescence microscope (Olympus BX51W1) and analyzed by ImageJ Fiji software (NIH, United States). The percentage of viable cells was quantitatively measured as ratio of the number of viable cells (detected in green) to the total number of cells (viable cells and dead cells, detected in red).

#### Adipogenic Differentiation

The adipogenic differentiation of 3T3-L1 pre-adipocytes was qualitatively investigated by Oil Red O staining of accumulated lipid droplets after 14 days of culture. The staining solution was prepared by dissolving 300 mg of Oil Red O powder in 100 ml isopropanol, subsequently diluted 2:3 in distilled water and sterile filtered. After 14 days of culture, samples (*n* = 4) were washed twice in TRIS-buffered saline (TBS)/CaCl_2_ 1×, fixed by submersion in 4% w/v paraformaldehyde for 30 min and washed again with TBS/CaCl_2_. After that, samples were incubated in Oil Red O staining solution for 30 min and washed twice with TBS/CaCl_2_ prior to observation by optical microscopy (Leica DFC290) at 5 and 20× magnification.

### Carrot-Derived Scaffolds for Bone Tissue Engineering

#### Mechanical Properties

The compression properties of control and decellularized carrot-derived scaffolds (*n* = 4, Ø = 10 mm, *t* = 4 mm) were tested by DMA, after immersion in PBS for 1 week (i.e., Δw plateau). A compression load was applied at a rate of 5% min^–1^ up to 60% strain, as previously reported for bone tissue engineering (Du et al., 2018). The elastic modulus was calculated as the slope in the 0–20% strain range from the obtained σ–ε curves. The stress at maximum strain and the area A under the σ–ε curves are also considered.

#### *In vitro* Direct Cytocompatibility Test

*In vitro* direct cytocompatibility test was performed using MC3T3-E1 cell line (ECACC No 99072810) as *in vitro* model for bone tissue engineering. Prior to seeding, scaffolds (*n* = 4) were coated with PLL and then transferred to new 24-well TCPS plates. Cells were seeded on scaffolds and 24-well TCPS plates (2 × 10^5^ cells per samples) following the above-stated procedure. Then, osteogenic differentiation was induced on samples (CA_osteo_) and wells (TCPS_osteo_) by culturing cells in osteogenic medium (M_osteo_: α MEM (M8042), 10% v/v FBS, 1% penicillin-streptomycin, 2 mM L-glutamine, 50 μg ml^–1^ ascorbic acid and 10 mM β-glycerophosphate) up to 14 days of culture; controls (CA_preosteo_, TCPS_preosteo_) were obtained by culturing cells in pre-osteoblast medium up to 14 days (M_preosteo_: αMEM (M8042), 10% FBS, 1% penicillin-streptomycin and 2 mM L-glutamine). Culture medium was renewed 24 h after seeding and, subsequently, every 2 days.

Metabolic activity of cells cultured on scaffolds and TCPS wells was assessed by alamarBlue assay at established time points (i.e., *t* = 1, 3, 7, 10, and 14 days), as previously described. Viable cells distribution was qualitatively and quantitatively investigated by LIVE/DEAD assay (*n* = 3) after 7 days of culture, as previously described.

#### Osteogenic Differentiation

Osteogenic differentiation of MC3T3-L1 cells seeded on decellularized carrot scaffolds (*n* = 4) was assessed by Alkaline Phosphatase (ALP) Assay (BioVision) after 14 days of culture. Briefly, samples were soaked in lysis buffer (1% w/v Triton X-100 and 50 mM HEPES) overnight. Lysates were transferred from each well to a new 96-well TCPS plate in triplicate; then, p-nitrophenyl phosphate (pNPP) solution was added to each well. The plate was incubated for 60 min to allow ALP enzyme to catalyze pNPP hydrolysis and the absorbance was measured by a spectrophotometer (λ = 405 nm). ALP activity was quantified by an ALP enzyme standard curve and normalized over DNA content. The amount of DNA was determined using SYBR Green I stain, with a calibration curve built by salmon sperm standards. Morphology of differentiation-induced and non-induced cells was observed by SEM analysis after 7 and 14 days of culture. Samples were fixed in 4% paraformaldehyde solution, washed three times with PBS and dehydrated by submersion in increasing ethanol concentrations (10% v/v: 10: 100% v/v, 10 min each step), lyophilized for 4 h and observed by SEM.

### Celery-Derived Scaffolds for Tissue Engineering of Tendons

#### Mechanical Properties

The tensile mechanical properties of control and decellularized celery-derived scaffolds (*n* = 4, Ø = 4 mm, *L* = 25 mm) were tested by DMA, after soaking samples in PBS for 1 week (i.e., Δw plateau). A tensile load was applied at a rate of 30% min^–1^ up to failure (Zitnay et al., 2018). The elastic modulus was calculated as the slope in the 0–5% strain range from the obtained σ–ε curves. Stress-strain curves were reported up to 20% strain (strain value reached by all the tested samples before failure); the stress at 20% strain and the area A under the stress-strain curve are also considered.

#### *In vitro* Direct Cytocompatibility Tests

*In vitro* direct cytocompatibility tests were performed on decellularized celery-derived scaffolds (Ø = 10 mm, *h* = 2 mm, *n* = 4). Mouse L929 fibroblastic cell line (ECACC No 85011425) was selected to investigate cell viability and contact guidance on celery structures. Cells were seeded on PLL-coated scaffolds and TCPS wells (2 × 10^5^ cells per well) following the above-stated procedure and cultured in M_fibr_ by renewing culture medium 24 h after seeding and, subsequently, every 2 days up to 14 days of culture.

Metabolic activity of cells cultured on scaffolds and TCPS wells was assessed by alamarBlue assay at established time points (i.e., *t* = 1, 3, 7, 10, and 14 days), while distribution of viable cells was qualitatively and quantitatively investigated by LIVE/DEAD assay after 7 days (*n* = 3), as described before.

Immunofluorescence microscopy was conducted on celery-derived scaffolds (*n* = 4) after 7 days of cell culture to assess actin microfilaments and nuclei alignment. Samples were washed in 1× TBS/CaCl_2_, fixed in 4% w/v paraformaldehyde for 30 min and washed again. Cells were permeabilized by submersion in 0.1% v/v Triton-X-100 for 15 min, soaked in 1% w/v bovine serum albumin (BSA) for 15 min, and washed three times in TBS/CaCl_2_. Actin staining was performed by incubating samples in phalloidin – FITC P5282 (1:1000 in 1% BSA, 45 min), while nuclei staining was performed with Hoechst 33258 (1 μg ml^–1^ in 1% BSA, 15 min). Samples were washed twice again and observed by fluorescence microscope (phalloidin: λ_em_ = 570 nm, Hoechst: λ_em_ = 460 nm). Results were analyzed by ImageJ Fiji software (NIH, United States): the preferential directionality was defined as the angle between the long axis of each cell and the preferential orientation (0°) of the observed cells. The percentage of aligned cells (0° ± 20°) over the total number of stained cells was then calculated.

### Statistical Analysis

Data are presented as mean ± standard deviation. Statistical analysis was performed by *t*-test to compare two data sets or by one-way ANOVA test, with Tukey’s multiple comparison test, to compare more data sets. GraphPad Prism 7 software was used; significance level was set at *p* < 0.05.

## Results

### Morphological and Physical Characterization

**FIGURE 2:**
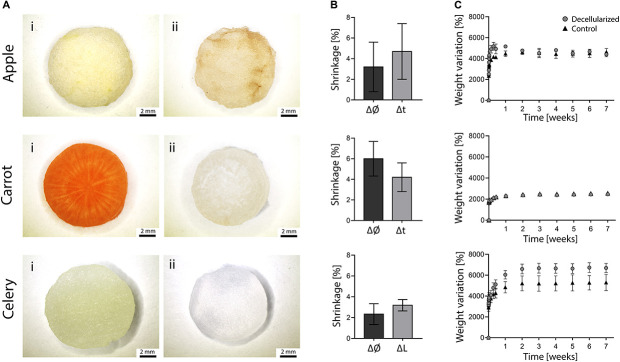
**(A)** Stereomicroscopy images of apple, carrot and celery – derived samples **(Ai)** prior to and **(Aii)** after decellularization (scale bar lower panel: 2 mm). **(B)** Dimensional variation of the diameter (Ø) and thickness (t) or length (L) of apple, carrot and celery samples after decellularization (*n* = 4). **(C)** Percentage weight variation of decellularized and control samples immersed in PBS at 37°C for 7 weeks (*n* = 4).

The morphology of plant tissues was investigated prior to and after decellularization to observe possible effects of the adopted protocol. All the considered plant tissues underwent a loss of pigmentation after the decellularization treatment (Figure 2Ai vs. Figure 2Aii), particularly noticeable in carrot samples due to the orange-colored appearance of the native tissue. The translucent, milky white appearance of the obtained treated samples is typical of decellularized structures, as widely described for both animal (Bühler et al., 2015; Fu et al., 2016) and plant-derived (Fontana et al., 2017; Gershlak et al., 2017) tissues.

Detected dimensional variations (Figure 2B) were assessed to be lower than 6% (i.e., maximum shrinkage registered for carrot-derived scaffolds), thus proving the absence of considerable volumetric changes and structural damage at the macroscale. This aspect is fundamental since possible changes in the dimension must be considered to design patient-specific and site-specific implantable scaffolds.

The weight variation of decellularized and control samples immersed in PBS at 37°C is shown in Figure 2C. Each sample absorbed water (i.e., 24 to 66 times their initial anhydrous weight) and reached a plateau value during the first week of immersion, after which the stable weights recorded up to 7 weeks proved the ability of the samples in retaining the absorbed fluid. In particular, stable weights were reached by apples after 24 h and by carrot and celery samples after 7 days of incubation. The weight variation at plateau for apple-derived and carrot-derived samples was 4500 and 2450%, respectively, with no difference comparing decellularized and native tissues (*p* > 0.05). The weight variation at plateau of celery-derived scaffolds was 6660%, with a significant increase in water absorption for decellularized samples compared to native tissue (*p* < 0.05). These data prove that the peculiar presence of high amount of water in plant tissues is preserved after decellularization and that the obtained structure is thus capable of fluids absorption, fundamental for the survival of cells in the tissue volume. In fact, water retention is a crucial aspect for the development of successful scaffolds able to substitute natural body tissues.

**FIGURE 3:**
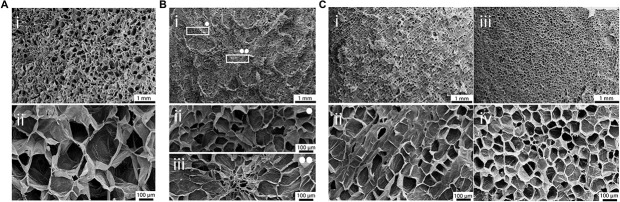
SEM micrographs of **(A)** apple, **(B)** carrot and **(C)** celery-derived scaffolds. **(A)** Decellularized apples, observed at (i) 15× and (ii) 100× magnification, show a homogeneous pores size and distribution. **(B)** Decellularized carrots present a non-homogeneous pores distribution **(Bi)**: round porosity and smaller pores are observed in the peripheral region (**Bii**, 100× magnification), oriented pores are observed in the central region (**Biii**, 100× magnification). **(C)** Decellularized celery were observed in both longitudinal (**Ci,Cii**) and transversal **(Ciii,Civ)** directions, to investigate their anisotropic structure. Scale bar upper panel: 1 mm; scale bar lower panel: 100 μm.

Scanning electron microscopy analysis was then performed on decellularized and control samples to investigate their structures at the microscale. A three-dimensional, highly porous structure is observed for all the considered plant tissues (Figure 3). No pore walls disruption or changes in the morphological structures were observed after the decellularization treatment (Supplementary Data 1), thus confirming that the used decellularization procedure (Hickey et al., 2018) allows for the preservation of the native morphology of the plant tissues. Apple-derived scaffolds (Figure 3Ai) show a homogeneous porosity, with roundish pores characterized by an average diameter of 420 ± 33 μm (Figure 3Aii). Differently, carrot-derived scaffolds were characterized by a heterogeneous structure. Thin channels, surrounded by radially oriented pores (70 ± 12 μm average size), run transversal to the sample section and constitute the central area of the xylem (Figure 3Bii). In the peripheral region, pores assume a round shape and a larger size (130 ± 26 μm average diameter), with no preferred orientation (Figure 3Biii). Finally, celery-derived samples were analyzed in both longitudinal (Figures 3Ci,ii) and transversal (Figures 3Ciii,iv) directions: the tissues appear to be constituted by packed pores (125 ± 11 μm average size), forming longitudinally oriented parallel channels. As typically detected in natural structures, both from mammalian and plant origin, intra-species small defects are often observed, such as irregularities in the surface morphology along the celery channels, such as changes in the channel diameter and path (i.e., bifurcations and convergences).

### *In vitro* Cytotoxicity

**FIGURE 4:**
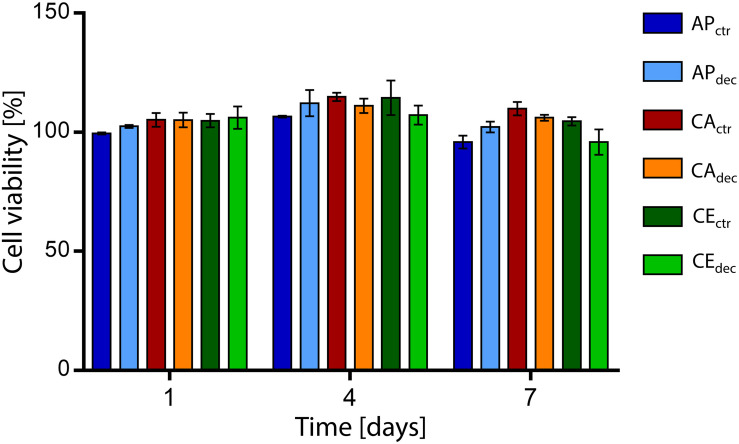
Percentage viability of L929 cells cultured with culture medium eluates obtained in contact with decellularized plant tissues (i.e., AP_dec_, CA_dec_, and CE_dec_) and control tissues (i.e., AP_ctr_, CA_ctr_, and CE_ctr_) for 1, 4, and 7 days (*n* = 3 per time point).

The percentage cell viability, expressed as the ratio of metabolic activity of cells cultured with eluates obtained from samples to the metabolic activity of cells cultured in control medium, is shown in Figure 4. The percentage cell viability is higher than 95% for each sample at each considered time point, confirming the absence of cytotoxic residues of the decellularization procedure in the vegetal structures and no cytotoxic effects caused by the chemical of the selected plants. Moreover, no statistical difference (*p* > 0.05) was detected comparing the viability of cells cultured in eluates obtained in contact with decellularized and control samples.

Since no cytotoxic effects were detected by *in vitro* indirect cytotoxicity tests, the suitability of the decellularized plant tissues for the regeneration of specific target human tissues was investigated by mechanical and *in vitro* direct cytocompatibility tests. In particular, according to their morphology and porosity, apple-derived scaffolds were investigated for applications in adipose tissue engineering for the isotropic structure, carrot-derived scaffolds for bone tissue engineering for the presence two different ranges of porosity in the same structure, and celery-derived scaffolds for the regeneration of tendons due to the possibility of vertically align cells in a tubular, anisotropic structure. Mechanical test procedures and consistent cell line models were selected according to the target tissue of each decellularized plant scaffold.

### Apple-Derived Scaffolds

Apple-derived scaffolds showed a relatively large and homogeneous porosity, suitable for adipose tissue regeneration. In fact, a pore size greater than 100 μm is adequate for an efficient provision of oxygen and nutrients in adipose tissue (AT) regeneration (Van Nieuwenhove et al., 2017) and it is widely described for other scaffolds developed for AT engineering [e.g., collagen-hyaluronic acid: (Van Nieuwenhove et al., 2017) 100–220 μm, polyurethane-based foams: (Gerges et al., 2018) 300–500 μm].

**FIGURE 5:**
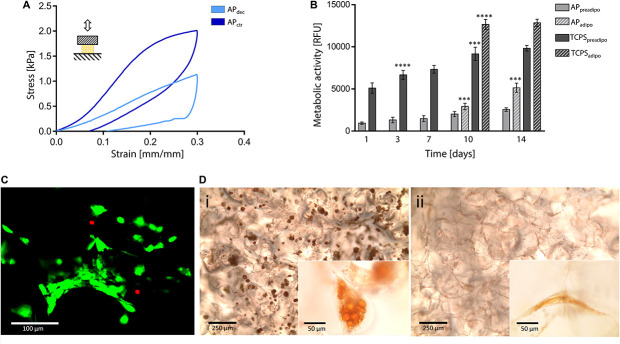
Decellularized apple for adipose tissue engineering. **(A)** Representative compression hysteresis cycle of apple-derived scaffolds and control samples. **(B)** Metabolic activity of 3T3-L1 preadipocytes cultured up to 14 days on apple scaffolds and TCPS plates. After 7 days of culture, cells were either induced toward adipogenic differentiation (i.e., AP_adipo_ and TCPS_adipo_) or cultured in pre-adipocyte medium (i.e., AP_preadipo_ and TCPS_preadipo_). *n* = 4 per time point, one-way ANOVA: ***p < 0.001, ****p < 0.0001 comparing the metabolic activity of the same sample at one time point to the previous time point. **(C)** Representative LIVE/DEAD staining of 3T3-L1 cells cultured on apple-derived scaffolds (scale bar: 100 μm). **(D)** Oil Red O staining of intracellular lipid droplets in **(Di)** differentiated and **(Dii)** non-differentiated 3T3-L1 cells cultured on apple-derived scaffolds. Differentiated cells are characterized by a round morphology and red-stained accumulated lipid droplets (scale bar: 250 μm; scale bar inset image: 50 μm).

To deeper investigate the suitability of this plant tissue for AT engineering, compression mechanical properties were tested. A representative hysteresis cycle of hydrated decellularized and control samples is shown in Figure 5A and calculated mechanical parameters are summarized in Table 1. Samples were tested up to 30% strain to replicate AT physiological conditions (Frydrych et al., 2015) and were able to sustain this deformation without failure. Hydrated decellularized apple-derived samples were characterized by a compression modulus of 4.17 ± 0.17 kPa; this is comparable to that of native human adipose tissue [e.g., E breast tissue (Van Nieuwenhove et al., 2017) = 2 kPa, E abdomen tissue (Omidi et al., 2014) = 3.3 kPa] and to those of recently proposed scaffolds for AT regeneration, including polyamidoamine foams (Rossi et al., 2016) (*E* = 3.4–4.4 kPa), decellularized AT (Yu et al., 2013) (*E* = 2.4–4 kPa) and collagen-hyaluronic acid scaffolds (Davidenko et al., 2010) (*E* = 5.39–6.73 kPa). No statistical difference was observed between the elastic moduli of decellularized and control samples (Table 1). An energy loss was observed during the unloading phase (Table 1), typical of the viscoelastic response that also characterizes native AT (Riaz et al., 2016). Moreover, the obtained H areas from apple-derived scaffolds are comparable to those of previously designed scaffolds (Chang et al., 2013; Contessi Negrini et al., 2019) for AT regeneration. A residual strain was observed in both control and decellularized samples at the end of the unloading phase (Table 1), as often observed in scaffolds for soft tissue engineering (Gao et al., 2014). Decellularized apple-derived scaffolds exhibit a decrease (*p* < 0.05) in stiffness k (i.e., calculated in the 15–20% strain) and maximum stress at 30% strain compared to untreated apple samples (Table 1), as experienced in previously developed plant-derived and organ-derived decellularized tissues [e.g., spinach-derived scaffold (Gershlak et al., 2017)]. Despite the lowered mechanical properties of apple-derived scaffolds, compared to the native tissues, the obtained mechanical properties correctly fit the range of those of native human AT, thus validating the adopted treatment for the production of tissue-mimicking scaffolds, in term of mechanical properties.

**TABLE 1:** Mechanical properties of decellularized (dec) apple (AP), carrot (CA), and celery (CE) tissues and non-decellularized tissues, as controls (ctr).

	Apple^χ^		Carrot^χ^		Celery^τ^	
	AP_dec_	AP_ctr_	CA_dec_	CA_ctr_	CE_dec_	CE_ctr_
Elastic modulus E [kPa]	4.17 ± 0.17	4.36 ± 0.02	43.43 ± 5.22*	83.48 ± 22.14*	594.78 ± 94.24	552.49 ± 12.44
Stiffness K [kPa]	4.33 ± 1.98*	9.47 ± 1.85*	−	−	−	−
Residual strain ε [%]	6.42 ± 0.08	6.48 ± 0.01	−	−	−	−
Maximum stress [kPa]	1.17 ± 0.28*	2.07 ± 0.05*	44.31 ± 8.59	51.49 ± 7.17	175.93 ± 40.96	174.60 ± 29.28
Hysteresis area H [J dm^–3^]	0.112 ± 0.023	0.153 ± 0.003	−	−	−	−
Area under the curve A [J dm^–3^]	−	−	11.1 ± 1.72	14.56 ± 2.30	15.37 ± 3.24	14.85 ± 1.75

Apple and carrot samples were tested by compression tests; celery samples were tested by tensile tests. *n* = 4, *t*-test: *p < 0.05. ^χ^Compression test; ^τ^tensile test.

*In vitro* direct cytocompatibility tests were performed using a pre-adipogenic cell line (3T3-L1). The metabolic activity of cells cultured on the apple-derived scaffolds increased throughout the 14 days of culture (Figure 5B), proving the ability of the apple scaffolds in sustaining pre-adipocyte cells growth and proliferation. The presence of viable cells adhered to the apple scaffold was also qualitatively proved by the LIVE/DEAD staining images (viability = 92.3 ± 4.9%, Figure 5C), where cells are shown to be distributed following the porous structure of the scaffold after 7 days of culture.

Adipogenic differentiation, induced after 7 days of culture, led to a rapid increase of metabolic activity for differentiated cells, compared to non-differentiated cells (Figure 5B, *p* < 0.05 differentiated vs. non-differentiated cells at the same time points) for both scaffolds and TCPS. Higher metabolic activity values were detected on TCPS wells compared to the scaffolds, which might be given by a higher cell seeding efficiency on the TCPS. In fact, apple-derived scaffolds were moved to new plates at each time point, to discharge cells adhered to the TCPS and not consider their contribution in the fluorescence signal. However, the percentage increase in metabolic activity (i.e., fluorescence value at 14 days vs. 1 day) was higher for cells cultured on the scaffolds than for those on TCPS (5.55-fold vs. 2.51-fold increase for differentiation-induced scaffolds and TCPS, respectively, and 2.70-fold vs. 1.92-fold increase for undifferentiated scaffolds and TCPS, respectively), evidencing a more efficient cell proliferation in the three-dimensional culture environment, compared to the traditional 2D plastic. After 14 days of culture, red droplets were observed by Oil Red O staining in differentiated cells cultured on apple-derived scaffolds (Figure 5Di), evidencing the intracellular lipid accumulation typical of adipocytes and an effective possible adipogenic differentiation of preadipocytes cultured on the apple scaffolds. Moreover, an increased size and roundness were observed in differentiated adipocytes compared to cells cultured in the pre-adipogenic medium (Figure 5Dii), typical morphology of adipocytes (Zoico et al., 2016).

### Carrot-Derived Scaffolds

The heterogeneous structure of carrot-derived scaffolds is characterized by radially oriented pores (70 ± 12 μm average size) in the central region and round larger pores (130 ± 26 μm average size) in the peripheral region, whose dimensions are comparable to the porosity of scaffolds designed for bone tissue regeneration (e.g., collagen/hydroxyapatite scaffold:(He et al., 2018) 50–100 μm, Na-alginate/hydroxyethylcellulose scaffold:(Tohamy et al., 2018) 89–217 μm, gelatin/alginate-coated β-TCP scaffold:(Pacelli et al., 2018) 141 ± 43 μm).

**FIGURE 6:**
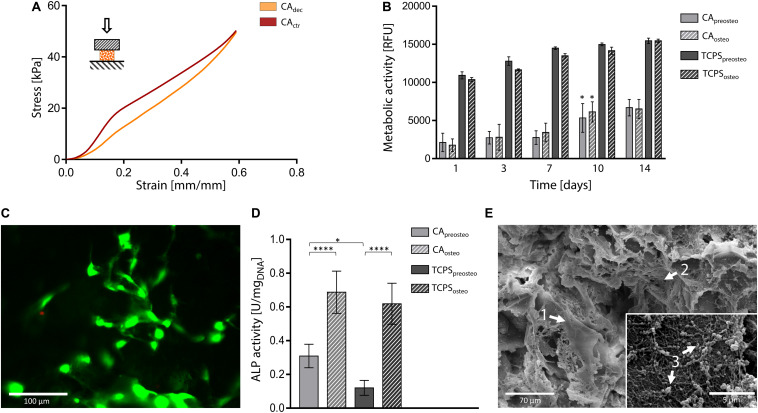
Decellularized carrot for bone tissue engineering. **(A)** Representative compression mechanical curve of carrot-derived scaffolds and control samples up to 60% strain. **(B)** Metabolic activity of MC3T3-E1 pre-osteoblast cultured up to 14 days on carrot scaffolds and TCPS plates. Cells were either induced toward osteogenic differentiation (i.e., CA_osteo_ and TCPS_osteo_) or cultured in pre-osteogenic medium (i.e., CA_preosteo_ and TCPS_preosteo_). *n* = 4 per time point, one-way ANOVA: *p < 0.05 comparing the metabolic activity of the same sample at one time point to the previous time point. **(C)** Representative LIVE/DEAD staining of MC3T3-E1 cells cultured on carrot-derived scaffolds (scale bar: 100 μm). **(D)** ALP activity of MC3T3-E1 cells culture on decellularized carrots and TCPS for 14 days (values are normalized over DNA content); *n* = 4 per time point, one-way ANOVA: *p < 0.05, ****p < 0.0001. **(E)** SEM micrographs of osteogenic-differentiated MC3T3-E1 cells (arrow 1) cultured for 14 days on carrot-derived scaffolds. Nano-fiber nets (arrow 2), attributable to ECM deposition, and inorganic extracellular secretion (arrow 3) can be observed and attributed to an on-going mineralization process (scale bar: 70 μm; scale bar inset image: 5 μm).

Compression tests were performed on both hydrated control and decellularized samples up to 60%. A representative stress-strain curve is shown in Figure 6A and calculated mechanical parameters are summarized in Table 1. No failure evidence was found in samples during the tests, evidencing that decellularized carrots can bear strains up to 60%. The obtained compression modulus of carrot-derived scaffolds is 43.43 ± 5.22 kPa, considerably lower than natural bone tissue (Bayraktar et al., 2004) (*E* = 3.5–18 GPa). However, mechanical properties comparable to those of carrot-derived samples have been widely proposed for non-load bearing bone scaffolds (e.g., calcium-silicate/chitosan scaffold *E* = 150–190 kPa; (Peng et al., 2019) PEG-dimethacrylate scaffold *E* = 225–300 kPa) (Chatterjee et al., 2010). Hence, a possible application for decellularized carrot-derived scaffolds could be bone fillers in non-load bearing conditions. Furthermore, an inorganic ECM deposition *in vitro* or *in vivo* by cells could improve the mechanical properties of the obtained scaffold over time, (Goyal et al., 2017) thus decreasing the mechanical gap between the native tissue and the scaffold.

*In vitro* direct cytocompatibility tests were conducted using a pre-osteoblast cell line (MC3T3-E1), by inducing osteogenic differentiation after cell seeding (Zamani et al., 2019). Metabolic activity resulted stable up to 7 days after seeding (Figure 6B). No statistical difference was observed (*p* > 0.05) between the metabolic activity of differentiation-induced and non-induced samples during the entire testing time, for both carrot-derived scaffolds and TCPS wells. An increase in the fluorescence values (*p* < 0.05) was then detected at day 10 in both differentiation-induced (i.e., CA_osteo_) and non-induced (i.e., CA_preosteo_) samples, then values remained stable up to 14 days of culture, with a 3.41-fold increase in cell metabolic activity from day 1 to day 14. The ability of the carrot scaffolds in sustaining pre-osteoblast cells growth and proliferation is therefore demonstrated. A plateau value in metabolic activity was reached after 7 days of culture on TCPS wells (i.e., TCPS_preosteo_ and TCPS_osteo_), with a slower cell growth rate when compared to carrot scaffolds (1.44-fold increase in metabolic activity from day 1 to day 14). Viable cells adhered to the decellularized carrot tissue were also qualitatively observed by LIVE/DEAD staining after 7 days of culture, with a percentage cell viability of 85.0 ± 7.2% (Figure 6C). ALP activity was investigated after 14 days of culture on carrot-derived scaffolds and TCPS plates (Figure 6D). Osteogenic differentiation was proved by an increased ALP activity (*p* < 0.05) for cells cultured in osteogenic medium (i.e., CA_osteo_ and TCPS_osteo_) compared to not induced cells (i.e., CA_preosteo_ and TCPS_preosteo_). After 14 days of culture, a uniform colonization was observed by SEM on both differentiation-induced (Figure 6E) and non-induced scaffolds (Supplementary Data 2). Cells uniformly colonized the pore walls of the carrot scaffolds, assuming an elongated morphology, typical of adhered cells (Figure 6E, arrow 1); nano-fiber three-dimensional nets were also observed (Figure 6E, arrow 2), which might be attributable to extracellular matrix deposition (López-Álvarez et al., 2011). Extracellular secretions were found on the surface of differentiation-induced cells (Figure 6E, arrow 3), evidencing an on-going mineralization as evidence in Thu et al. (2017). In fact, the nano-sized particles detected for carrots structures could be possibly attributed to apatite crystals or bone sialoprotein, a osteogenic marker associated to mineralization, as experienced in previous studies where MC3T3-E1 cells were seeded on collagen scaffolds (Cao et al., 2015).

### Celery-Derived Scaffolds

Celery-derived scaffolds are characterized by a longitudinally oriented structure, constituted by 125 ± 11 μm size pores aligned to form parallel channels. Scaffolds with an oriented morphology are particularly suitable for cell alignment and to mimic the structure of native anisotropic connective tissue of tendons (Gurkan et al., 2010). Due to their oriented structure, celery-derived scaffolds were therefore subjected to mechanical and *in vitro* biological tests to assess their potential adequacy as scaffolds for tendon regeneration.

**FIGURE 7:**
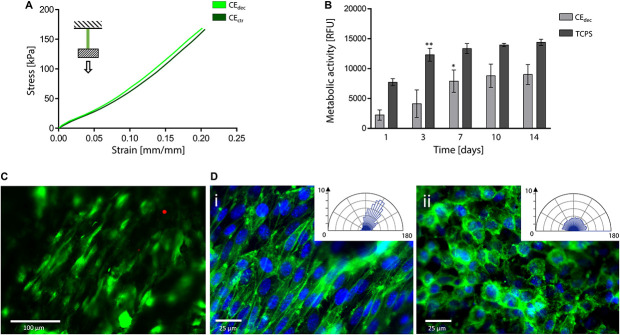
Decellularized celery for tendon/ligament engineering. **(A)** Representative tensile mechanical curve of decellularized and control celery samples. **(B)** Metabolic activity of L929 cells seeded on celery-derived scaffolds and TCPS wells up to 14 days of culture. *n* = 4 per time point, one-way ANOVA: *p < 0.05, **p < 0.01 comparing the metabolic activity of the same sample at one time point to the previous time point. **(C)** Representative LIVE/DEAD staining of L929 cells cultured on celery-derived scaffolds (scale bar: 100 μm). **(D)** Representative immunofluorescence microscopy images of aligned **(Di)** and randomly organized **(Dii)** cells after 7 days of culture on celery scaffolds. Nuclei are stained in blue (Hoechst 33258), while actin microfilaments are stained (phalloidin – FITC) in green (scale bar: 25 μm). Polar histograms of the cell directionality are depicted in the inset boxes.

Tensile tests were performed to investigate the mechanical properties of control and decellularized samples. Representative σ–ε curves are shown in Figure 7A and in Supplementary Data 3, while calculated mechanical parameters are summarized in Table 1. The samples were able to withstand 20% strain without failure, corresponding to the maximum value of deformation to which natural tendons are subjected *in vivo* (Mathew et al., 2012). The elastic modulus E of the decellularized celery scaffolds is equal to 0.59 ± 0.09 MPa, with no statistical difference (*p* < 0.05) compared to control. Despite the higher stiffness registered in native tendons (*E* = 500–1200 MPa), (Islam et al., 2017) scaffolds with relatively low elastic modulus have also been designed (e.g., Agar-PVA hydrogel *E* = 0.12–2.3 MPa;(Sabzi et al., 2017) silk fibroin scaffolds *E* = 1.2–1.4 MPa;(Font Tellado et al., 2017) GelMA-alginate hydrogel *E* = 5–9 kPa), (Rinoldi et al., 2019) proving the potential of celery-derived scaffolds for the regeneration of low-loaded anatomical regions, such as in hand tendons [physiological loads of 3–24 N were registered in human hand flexor tendons, (Edsfeldt et al., 2015) while 920–1510 N loads were detected in Achilles tendons] (Taylor and Arnold, 2016). The tensile strain at break was 23.9 ± 2.5 and 27.8 ± 3.5% and the tensile stress at break was 200.5 ± 49.9 and 246.1 ± 15.4 kPa for CE_dec_ and CE_ctr_, respectively. However, samples rupture occurred in correspondence of the clamps, which might result in an underestimation of the obtained values at break.

*In vitro* direct cytocompatibility was investigated using L929 fibroblast cell line. The metabolic activity (Figure 7B) increased until a plateau value was reached (*t* = 7 days of culture on celery-derived scaffolds, *t* = 3 days of culture on TCPS wells). Then, the detected fluorescence values were stable for the entire testing time (*t* = 14 days), suggesting cell confluence. Despite the higher metabolic activity on TCPS wells, the percentage increase of metabolic activity at *t* = 14 days compared to *t* = 1 day was significantly higher on celery-derived scaffolds, evidencing an efficient cell growth on the scaffolds (4.03-fold increase in celery-derived scaffolds vs. 1.86-fold increase on TCPS wells). The LIVE/DEAD staining confirmed the presence of viable L929 cells adhered to the decellularized celery scaffolds (Figure 7C, percentage cell viability equal to 84.5 ± 7.2%).

Cell alignment was also observed in several regions of the celery surface by LIVE/DEAD staining and was further investigated by immunofluorescence assay. Nuclei were stained with Hoechst 33258, while actin microfilaments were stained with phalloidin – FITC. In several regions of the scaffold surface, corresponding to the previously observed well-defined areas in the scaffold morphology (Figure 3Cii), a preferential cell orientation was evidenced and more than 60% of the cells were assessed to have a <20° alignment angle (Figure 7Di). This result is comparable to the aligned cells percentage observed on previously developed oriented scaffolds (Schoenenberger et al., 2018; Wang et al., 2018) addressed to tendon tissue engineering. Randomly organized regions were also detected, where cells are characterized by a round morphology with no preferential orientation (Figure 7Dii). The presence of both aligned and random cells is consistent with the structure of native tendons, characterized by bundles of aligned collagen fibers surrounded by soft interfascicular matrix, with a less defined and oriented structure (Thorpe et al., 2016).

## Discussion

The use of decellularized plant tissues represents a valid alternative in the development of three-dimensional scaffolds for tissue engineering applications. However, their application for tissue engineering purposes [i.e., cardiac tissue regeneration, (Gershlak et al., 2017) muscle regeneration] (Modulevsky et al., 2014) is still poorly investigated, (Modulevsky et al., 2014) focused on few 3D tissues, such as apple, (Modulevsky et al., 2016) or limited to 2D plant tissues, such as leaves (Gershlak et al., 2017). Moreover, most pioneer works published so far on the use of decellularized plant tissues aim at demonstrating the suitability of plant tissues as potential scaffolds, (Modulevsky et al., 2016; Fontana et al., 2017; Adamski et al., 2018; Dikici et al., 2019) with only few examples targeting at the regeneration of specific tissues (Gershlak et al., 2017; Lee et al., 2019; Cheng et al., 2020). The decellularized plants microarchitectures can mimic the complexity of native human tissues, with no need of multi-step material processing associated to the majority of natural and synthetic polymer derived scaffolds (Jammalamadaka and Tappa, 2018; Zhang et al., 2019). Plants can be considered an alternative source of scaffolds for their low cost, high and easy availability and the absence of ethical issues compared to animal and human-harvested decellularized tissues. Moreover, as animal and human sources are often associated to an important interpatient variability and to the possible transmission of pathogenic agents, (Gilbert et al., 2006; Song and Ott, 2011) the adoption of plant sources may constitute a more reliable path for the obtainment of commercially suitable scaffolds. However, compared to mammalian-derived decellularized tissues, plant tissues are composed by relevant percentages of cellulose, which is not degradable or very slowly degradable in humans, so that cellulose modifications are under investigation to promote cellulose-based bioresorbable scaffolds degradation (Novotna et al., 2013). Thus, considering the plant-derived scaffolds here proposed, their use has potentially to be referred to as biostable scaffolds that, after implantation, integrate with the surrounding tissues and promote cells and tissues colonization (Hickey and Pelling, 2019). In fact, our data show stability in PBS at 37°C up to 7 weeks (i.e., duration of the test), which are in line with previously published data demonstrating absence of apple scaffolds degradation subcutaneously implanted *in vivo* that, however, showed a successful integration with the surrounding tissues (Modulevsky et al., 2016).

Previous studies (Modulevsky et al., 2014, 2016; Fontana et al., 2017; Gershlak et al., 2017; Hickey et al., 2018) on plant-derived scaffolds (e.g., spinach leaves, parsley, apple hypanthium) assessed the cytocompatibility, biocompatibility and pro-angiogenic properties of the decellularized plant structures even in the absence of a pre-existing vascular network, opening up a broad range of possibilities for these emerging biomaterials. However, the potential in tissue engineering associated to the plant kingdom is still to be deeply and fully investigated: the wide variety of sizes, textures, structures and compositions offered by nature may allow for the targeting of specific human tissues to be regenerated, depending on the peculiar mechanical, physical and morphological features requested for the specific application. Three different plant tissues (i.e., apple hypanthium, carrot xylem, and celery stem) were here selected for the production of decellularized scaffolds to address site-specific human tissues regeneration. The large-size and round porosity adequate for adipocyte colonization and the isotropic structure of apple hypanthium present interesting similarities with human AT, and mechanical investigation confirmed its suitability in mimicking the mechanical features of the native AT (E_apple_ = 4 kPa vs. E_adipose tissue_ = 2–20 kPa) (Omidi et al., 2014; Van Nieuwenhove et al., 2017). The decellularized scaffolds also show mechanical features comparable to those of other AT scaffolds proposed in literature (Davidenko et al., 2010; Yu et al., 2013; Rossi et al., 2016). Carrot-derived scaffolds are characterized by a smaller and heterogeneous porosity, related to higher mechanical properties and lower water adsorption, compared to the apple structures. In particular, the average pore size found in carrot samples is comparable to previously developed scaffolds (He et al., 2018; Pacelli et al., 2018; Tohamy et al., 2018) for bone tissue engineering. Thus, decellularized carrot tissue was addressed to bone tissue engineering and, according to the obtained mechanical properties (E_carrot_ = 43.43 ± 5.22 kPa), non-load bearing applications were selected, such as maxillo-facial and cranial bone regeneration. In fact, despite the superior mechanical features of natural bone (e.g., *E* = 25–240 MPa for mandibular trabecular bone), (Lakatos et al., 2014) the obtained carrot-derived scaffolds have mechanical properties comparable to other proposed scaffolds for the regeneration of non-load bearing bone tissue (*E* = 150–300 kPa) (Chatterjee et al., 2010; Peng et al., 2019). Finally, celery stems are characterized by an anisotropic structure, constituted by packed pores organized in parallel longitudinal channels. This peculiar architecture, functional in nature for water transport along the stem to the leaves and maintained after the decellularization treatment, is fundamental for the development of successful scaffolds, since it would allow to nutrients and fluids transport. Due to its oriented structure, celery was selected for the regeneration of naturally anisotropic tissues (i.e., tendons); in particular, morphology and size of samples (Ø = 4 mm, *L* = 25 mm) are comparable to the natural size of hand tendons (e.g., *palmaris longus*, Ø = 4–4.5 mm) (Ito et al., 2001). Mechanical tests demonstrated the capability of celery samples to withstand the physiological deformation to which tendons are subjected *in vivo*, (Mathew et al., 2012) thus proving their mechanical adequacy. The elastic modulus (E_celery_ = 0.59 ± 0.09 MPa) is comparable to other proposed scaffolds for similar applications (*E* = 0.12 – 2.3 MPa), (Font Tellado et al., 2017; Sabzi et al., 2017) despite being lower than those of natural tendon tissue (E_tendon_ = 500–1200 MPa) (Islam et al., 2017).

The produced decellularized plant tissues were able not only to support cells adhesion and proliferation, but also to sustain a correct functionality of cells for the specific tissue to be regenerated, as proved by the conducted *in vitro* tests, fundamental for the regeneration of functional tissues. For instance, lipid droplets accumulation was demonstrated on apple scaffolds for adipose tissue engineering, increased ALP activity and inorganic matrix deposition was assessed on carrot scaffolds for bone tissue engineering, and preferred orientation of cells was observed for celery samples for anisotropic tissue regeneration (e.g., tendon). Finally, we adopted a previously published protocol for plants decellularization, (Hickey et al., 2018) and we qualitatively checked the decellularization as a change in color of the decellularized scaffolds (Gershlak et al., 2017). However, more detailed tests will be required to quantitatively assess the effective decellularization of the proposed plants that, given the different structures, might respond differently to the adopted decellularization procedure and the protocol could be adapted for a more efficient decellularization, as recently demonstrated by the use of DNAse (Phan et al., 2020). Despite these tests and further studies on the *in vivo* biocompatibility, degradation or integration with the surrounding tissues of the here proposed scaffolds are required, the obtained results suggest the potential versatile use of plant-derived scaffolds for different tissue engineering purposes.

## Conclusion

The potential of decellularized apple, carrot and celery-derived tissues as scaffolds for the regeneration of adipose tissue, bone tissue and tendons, respectively, is here demonstrated. Adequate morphological, physical and mechanical features were obtained for the decellularized scaffolds toward the specific tissues to be regenerated. Furthermore, *in vitro* tests proved the ability of the decellularized plant tissues in supporting cells adhesion, proliferation and functionality, fundamental for the regeneration of the selected functional human tissues. Despite further studies are required to verify the feasibility of *in vivo* implant of decellularized plants and their integration with the host organism, the results here presented suggest a possible use of different plant tissues for versatile tissue engineering applications.

## Data Availability Statement

All datasets presented in this study are included in the article/Supplementary Material.

## Author Contributions

The manuscript was written through contributions of all authors. All authors have given approval to the final version of the manuscript.

## Conflict of Interest

The authors declare that the research was conducted in the absence of any commercial or financial relationships that could be construed as a potential conflict of interest. The handling editor LR declared a past co-authorship with several of the authors SF and NC.
