# Supplementary material for: Plant Tissues as 3D Natural Scaffolds for Adipose, Bone and Tendon Tissue Regeneration
Source: Front Bioeng Biotechnol. 2020 Jun 30;8:723. doi: 10.3389/fbioe.2020.00723 (PMC7344190; doi:10.3389/fbioe.2020.00723)
Supplement: Supplementary file 1 [file Image_1.pdf]

Plant Tissues as 3D Natural Scaffolds for Adipose, Bone and Tendon Tissue Regeneration

Supplementary Material

Supplementary Data

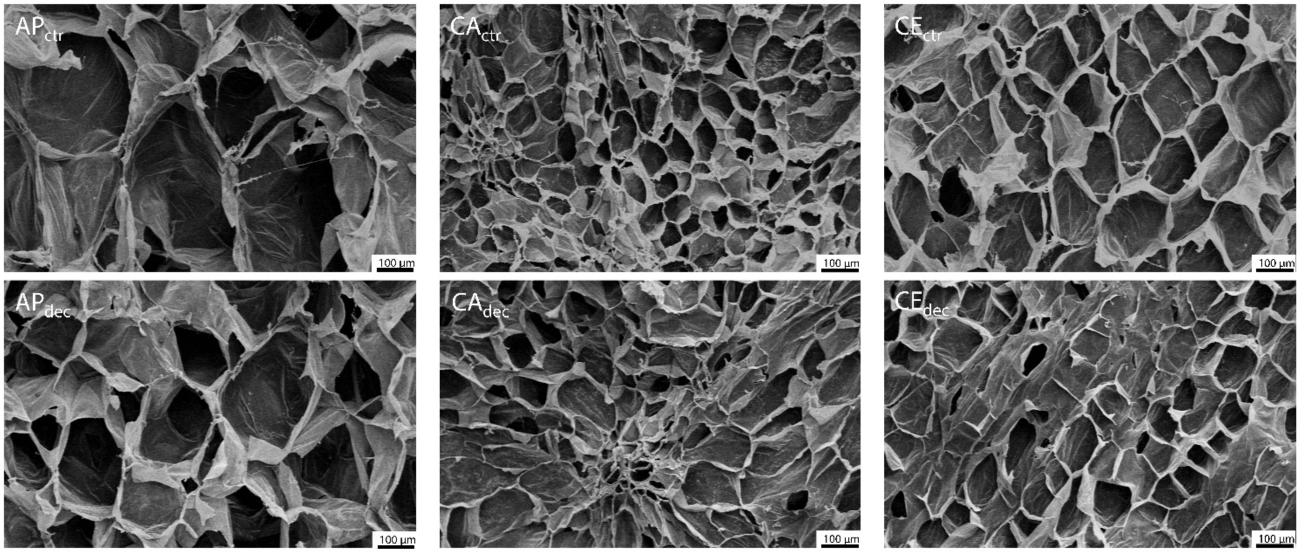

Supplementary Data 1: SEM micrographs of apple (AP_{ctr}), carrot (CA_{ctr}) and celery (CE_{ctr}) in comparison to decellularized apple (AP_{dec}), carrot (CA_{dec}) and celery-derived (CE_{dec}) scaffolds; scale bar: 100 µm.

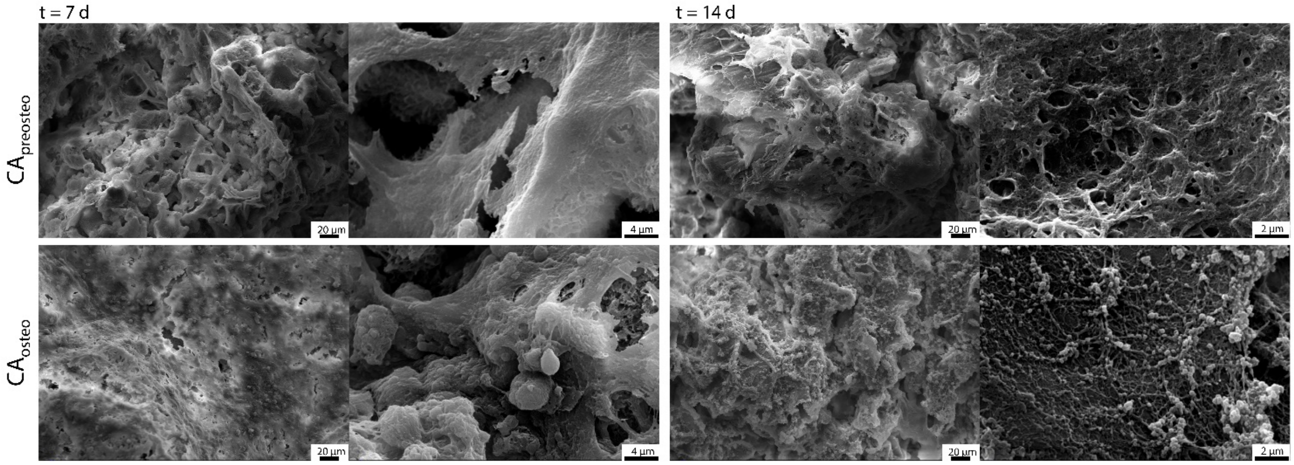

Supplementary Data 2: SEM micrographs of osteogenic-differentiated (CA_{osteo}) and non-differentiated ($CA_{preosteo}$) MC3T3-E1 cells cultured for t = 7 and 14 days on carrot-derived scaffolds. After 14 days of culture, a uniform colonization was observed on both differentiation-induced and non-induced scaffolds. Scale bar left micrographs: 20 µm, scale bar right micrographs: 4 µm (t = 7 d) and 2 µm (t = 14 d).

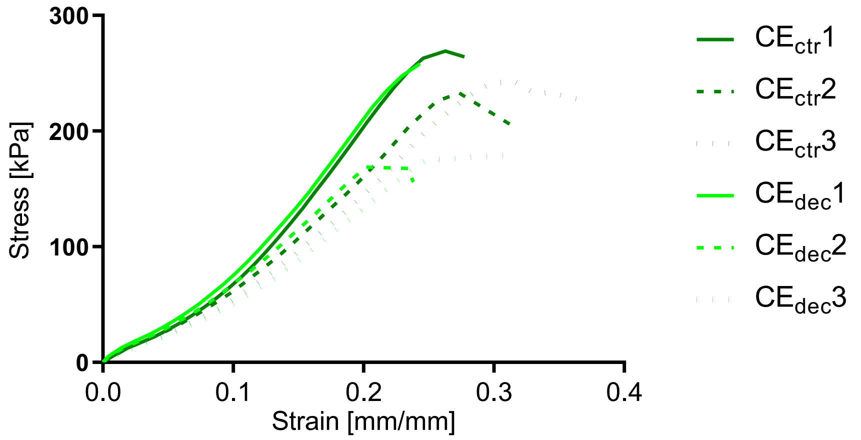

Supplementary Data 3: Representative tensile mechanical stress/strain curves at failure of decellularized (CE_{dec}) and control (CE_{ctr}) celery samples.
